# *Ganoderma lucidum* Ameliorates Neurobehavioral Changes and Oxidative Stress Induced by Ethanol Binge Drinking

**DOI:** 10.1155/2020/2497845

**Published:** 2020-07-30

**Authors:** Chirlene Pinheiro Nascimento, Diandra Araújo Luz, Carla Cristiane Soares da Silva, Cláudia Marques Rosa Malcher, Luanna Melo Pereira Fernandes, Herta Stutz Dalla Santa, Antônio Rafael Quadros Gomes, Marta Chagas Monteiro, Carolina Heitmann Mares Azevedo Ribeiro, Enéas Andrade Fontes-Júnior, Cristiane Socorro Ferraz Maia

**Affiliations:** ^1^Faculty of Pharmacy, Institute of Health Sciences, Federal University of Pará, Belém, 66075-900 Pará, Brazil; ^2^State University of Central-West, Department of Food Engineering (DEALI), Cedeteg Campus, Vila Carli, R. Simeão Varela de Sá, Guarapuava, 85040-080 Paraná, Brazil; ^3^Laboratory of Immunology, Pharmacy Faculty, Institute of Health Science, Federal University of Pará, Belém, PA, Brazil

## Abstract

*Ganoderma lucidum*, mushroom used for centuries by Asian peoples as food supplement, has been shown interesting biological activities, including over the Central Nervous System. Besides, these mushroom bioactive compounds present antioxidant and anti-inflammatory activities. On the side, binge drinking paradigm consists of ethanol exposure that reflects the usual consumption of adolescents, which elicits deleterious effects, determined by high ethanol consumption, in a short period. In this study, we investigated whether the Aqueous Extract of *G. lucidum* (AEGl) reduces the behavioral disorders induced by alcohol. Male (*n* = 30) and female Wistar rats (*n* = 40), seventy-two days old, were used for behavioral/biochemical and oral toxicity test, respectively. Animals were exposed to 5 binges (beginning at 35 days old) of ethanol (3 g/kg/day) or distilled water. Twenty-four hours after the last binge administration, animals received AEGl (100 mg/kg/day) or distilled water for three consecutive days. After treatment protocol, open field, elevated plus maze, forced swim, and step-down inhibitory avoidance tests were performed. Oxidative stress parameters were measured to evaluate the REDOX balance. Our results demonstrated that AEGl elicited the recovery of spontaneous horizontal exploration capacity, anxiogenic- and depressive-profile, as well as short-term memory damage induced by binge-ethanol exposure. The behavioral effects of the extract were associated to the reequilibrium of the animals' REDOX balance. Thus, AEGl, a medicinal mushroom, ameliorates behavioral alteration on a model of motor, cognitive and psychiatric-like disorders induced by binge drinking paradigm and emerges as a useful tool as a food supplement in the management of disorders of alcoholic origin.

## 1. Introduction

Ethanol is a psychoactive drug able to alter several body systems, including the central nervous system (CNS). The exact molecular mechanisms associated to these injuries are still poorly understood, even though studies indicate the involvement of the certain mechanisms, such as the neuroinflammation and oxidative stress, on the neuropathological processes [1–4]. The injury mechanisms seem to be related to the pattern of consumption [5, 6]. Binge drinking is a pattern that consists of acute intoxication, i.e., 5 or more doses for men and 4 or more doses for women, during a short period of 2 hours, approximately, reaching a minimum blood alcohol concentration (BAC) of 80 mg/dL [6–8]. Previous researches carried out by our group have demonstrated that binge drinking pattern during adolescence modifies behavioral profiles, as spontaneous locomotion and motor coordination, as well as displays psychiatry-like and cognitive disorders, as anxiogenic and depressive phenotype, and memory impairment, associated to reduction-oxidation reactions (REDOX) imbalance in rodents [9, 10].

The knowledge of the probable mechanisms that underlie the ethanol tissue damage provides critically the search for alternatives managements to reduce these injuries, since there is still no adequate therapeutic management [11]. A promising alternative would be the use of therapeutic supplementation that consists of the use of bioactive functional foods, since they offer lower health risks and intoxication [12–14].

Actually, the use of dietary supplementation has increased, focused on reducing the onset or even treat diseases such as cancer, cardiovascular diseases, and neurological disorders ([12]., [14, 15]). Some studies support the use of functional products for neurochemical changes associated to cognitive improvement, mood, and aging [12, 15]. In this regard, the mushroom *Ganoderma lucidum* (W. Curt.:Fr.) P. Karst. (Ganodermataceae, Agaricomycetes) emerges as an important source of bioactive compounds that can be used in the diet as supplementation, aiming to improve health [16, 17]. Known as the “elixir of life” or “mushroom of immortality,” officially used as a dietary supplement, this mushroom presents popular indications for “improving memory,” “retarding senility,” among other purposes [18–21]. Bioactive compounds such as triterpenes, lectins, and polysaccharides have been described in their composition [21–24].

A recent study reported that *G. lucidum* is able to act on free radicals and suppress lipid peroxidation, normalizing the balance between antioxidant defenses and reactive oxygen species, thus reducing oxidative stress [19]. In fact, studies have confirmed therapeutic activities of *G. lucidum* in the CNS, such as antidepressant action, cognitive improvement, and hypnotic properties, but the mechanisms have not yet been elucidated [22, 24].

In this sense, we hypothesize that pharmacological and biochemical activities from bioactive compounds of natural products, as *G. lucidum*, may diminish behavioral alterations induced by alcohol disorders. Thus, this work aims at evaluating the behavioral responses and the oxidative balance of *G. lucidum* in adult rats submitted to the binge drinking paradigm.

## 2. Material and Methods

### 2.1. Animals

Male Wistar rats, thirty-five days old at the beginning of the experiment, were used for behavioral/oxidative stress analyses. Female Wistar rats, seventy-two days old, were used for oral toxicity test. The animals were maintained at collective cages (5 animals per cage) to avoid stress by isolation and kept in controlled conditions of temperature (23 ± 1°C), exhaustion, light/dark cycle (12 h; lights ON 7 AM), water, and food *ad libitum*. All procedures were approved by the Ethics Committee on Experimental Animals of the UFPA (BIO-CEPAE 09-15), in accordance with the criteria established by the NIH Guide for the Care and Use of Laboratory Animals.

### 2.2. Preparation of Aqueous Extract of *Ganoderma lucidum* (AEGl)

The lyophilized *G. lucidum* mycelium, provided by BRASMICEL-SP was deposited on the Micoteca of the Laboratório de Bioprocessos de Cogumelos—DEALI (UNICENTRO, Guarapuava, Paraná, Brazil) and triturated, mixed with distilled water and homogenized in the vortex for five minutes. The resulting suspension was filtered and administered orally at a final volume of 0.1 ml for each 100 g of animal weight, equivalent to 100 mg/kg/day [25].

### 2.3. Binge Drinking Paradigm and AEGl Treatment

**Figure 1:**
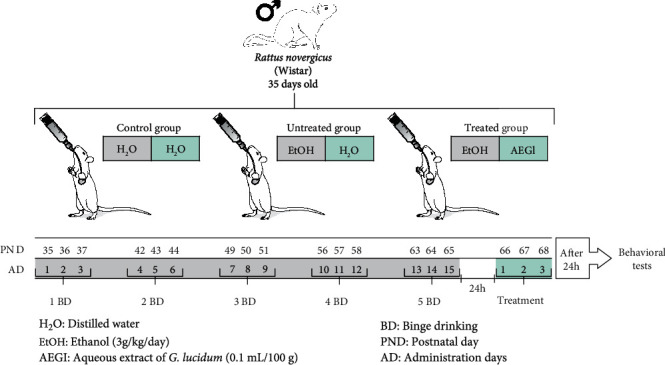
Experimental design of the study.

All administrations were performed by oral gavage with orogastric cannula (Insight, Brazil). It was classified as binge the pattern of three followed administrations of ethanol per week, once per day [9]. Three groups were used: control, untreated, and treated individuals. The treated and untreated groups were exposed to 5 binges (beginning at 35 days old) of ethanol (3 g/kg/day; 20% *w*/*v*), which consists the middle adolescence until the adult phase [10]. This binge-drinking paradigm reaches a BAC of approximately 250 mg/dL [10], which reflects the binge-drinking pattern (over 80 mg/dL; [8]). Twenty-four hours after the last binge administration, animals received AEGl (treated) or distilled water (untreated) for three consecutive days (Figure 1).

### 2.4. Oral Toxicity Assay

The oral toxicity test was based on OECD Guideline 420, with adaptations. Female Wistar subjects were divided into four groups. The test groups (*N* = 15/group) received orally an acute dose of 2,000 mg/kg or 5,000 mg/kg of *G. lucidum*, while the control group (*N* = 5 animals/group) received distilled water in an equivalent volume. Animals were evaluated as predicted by the Behavioral Pharmacological Triage [26]. Animal mortality was observed after 30 min, 1 h, 2 h, and 24 h of administration. These observations were maintained up to 14 days after the administration of the *G. lucidum*, once a day and always at the same time, for 30 minutes. Following behavioral toxicity evaluation, animals were sacrificed by cervical displacement followed by cardiac puncture for blood collection and hematological and oxidative biochemistry analysis on the first day (D1) and other groups on the fourteenth day (D14) after protocol administration.

### 2.5. Behavioral Evaluations

Previously to behavioral tests, animals were conducted for the behavioral experimental room for habituation for 2 h that consists of a low-intensity light (12 lx) and sound-attenuated room. All experimental procedures were carried out between 9 : 00 AM and 4 : 00 PM.

#### 2.5.1. Open Field Test (OF)

To perform the test, a black acrylic arena (100 x 100 cm wide and 40 cm high) was used. Briefly, animals were placed individually on the center of the apparatus, and free movement was allowed for 5 minutes. The test was videotaped for further analysis by ANY-maze TM (Stoelting, USA). The motor parameters (i.e., total distance traveled and number of rearing) were measured [9].

#### 2.5.2. Elevated Plus Maze (EPM) Test

Animals were submitted individually to an elevated wooden apparatus 50 cm from the floor, with two enclosed arms and two open arms (50x10 cm) opposite each other. In the closed arms, there are lateral walls of 40 cm of height. Each animal was placed on the center of the EPM facing one of the closed arms, being allowed to explore the equipment for 5 minutes. The percentage of time spent in the open arms (%OAT), the percentage of open arms entries (%OAE), and the number of entries in the closed arms (EAE) was counted. The %OAE and %OAT were calculated according to the formula [(open/total) × 100]. An entry was counted whenever the animal placed four paws in an arm of the maze [9].

#### 2.5.3. Forced Swimming (FS) Test

The apparatus of the forced swimming test (FS) consists of a 50 cm high and 30 cm diameter cylinder containing 40 cm of water column at a temperature of 23 ± 1°C. Animals were placed on the center of the equipment for 5 minutes. The initial 2 min consisted of the habituation phase. In the final three minutes, immobility time (brief movements to keep floating) was measured [9, 27].

#### 2.5.4. Step-Down Inhibitory Avoidance (IA) Test

The test was carried out in an aluminum box (50 x 50 x 35 cm), containing one safe platform and a floor consisting of 15 parallel copper bars connected to an electric stimulator. Briefly, the animals were habituated for 180 seconds to the apparatus, allowing free exploration. After 24 hours, the animals were submitted to the training session and reexposed to the safe platform of the apparatus. Immediately after the animal step-down from the platform with the four legs on the grids, an electric shock of 0.4 mA for 1 second (aversive stimulus) was applied. Each animal was reexposed again 1.5 h after the training to verify short-term memory acquisition. Thus, the latency to step-down from the secure platform was recorded as the level of short-term memory retention [9].

### 2.6. Oxidative Biochemistry Assays

#### 2.6.1. Determination of Plasma Malondialdehyde (MDA) Concentration

MDA dosage was based on the formation of the complex with thiobarbituric acid with MDA. The TBA was prepared according to enterprise guideline (Sigma Aldrich, Germany). Briefly, 1 mL of TBA solution (10 nM) plus 500 *μ*L of the plasma sample was mixed. The TBA-MDA complex levels were detected by the spectrophotometer device (wavelength 535 nm) [28].

#### 2.6.2. Measurement of Trolox Equivalent Antioxidant Capacity (TEAC)

TEAC evaluation consists of measuring the inhibition of the ABTS+• cation (2,2-Azinobis- [3-ethylbenzothiazoline-6-sulphonate radical, diammonium salt) in the sample. Briefly, 2970 *μ*L of working solution was added to the cuvette, followed by reading the absorbance spectrophotometer. This value corresponds to T0. Subsequently, 30 *μ*L of the homogenized plasma sample was added and the timer was activated to mark 5 minutes. After 5 minutes, the absorbance, corresponding to T5, was recorded [29]. The calculation of the results was obtained from the use of formulas based on the absorbance results of the A-F tubes, curve standard (*R*^2^ = 0.9964; *y* = 0.01732 x − 0.1367) to obtain the calculation of the Trolox equivalent sample, in the following order: TAAc = (T0 + T5/T0) − White control TAA and TEAC = (TAAc–0.1367)/0.01732.

The total antioxidant potential of serum determined as *μ*AC/mL TEAC and calculated by a calibration curve with different amounts of Trolox [29, 30].

#### 2.6.3. Measurement of Superoxide Dismutase (SOD) Activity

SOD dosage was based on the competition of SOD with XOD (xanthine oxidase enzyme) by the superoxide radical. A 100 *μ*L aliquot of each blood sample was mixed with 400 *μ*L of ice water to promote hemolysis. The volume of 3 mL was taken for reading in the cuvettes by a spectrophotometer (550 nm) in the time of 5 minutes. The absorbance values were recorded from time 0 (T0) and time 5 (T5). T0 was noted before the addition of the XOD reagent, then, the XOD was added and counted for 5 minutes to check the final reaction (T5). The final calculation considered: SOD = (%inhibition) x (Dilution factor)/(50%) x (0.10), which 50% is the reduction rate of Cytochrome C by unit definition; and 0.10 the volume in milliliters of the sample used in each test [31]. Final results were expressed in units/mL.

#### 2.6.4. Measurement of Total Glutathione (GSH)

GSH analysis was based on the ability of the GSH present in the sample in reduce 5,5-dithiobis-2-nitrobenzoic acid (DTNB) to nitrobenzoic acid (TNB). The sample was read by the spectrophotometer at 412 nm of wavelength. After hemolysis generated by the mixture of 20 *μ*L of the total blood and 180 *μ*L of distilled water, 20 *μ*l of the hemolysate was added in 20 *μ*l of distilled water plus 4 *μ*L of PBS/EDTA and vortexed. An aliquot of 3 mL of the sample was placed in the spectrophotometer reading cuvette, and the absorbance at time 0 (T0) was measured. Then, 100 *μ*L of the DTNB was added and the time count was started for 3 minutes. The reading time of 1 minute (T1) and 3 minutes (T3) from the addition of the DTNB was recorded [32]. To calculate the GSH concentration in the sample, the equation of a standard curve previously performed (*y* = 154.38x − 3.2983; *R*^2^ = 0.9421) was used. The concentrations of GSH in the samples were expressed in *μ*g/mL.

### 2.7. Statistical Analyses

Results are expressed as mean ± S.E.M. of *n* = 6 − 10 animals/group for behavioral analysis and *n* = 4–7/group for oxidative biochemical assays. Statistical comparison was performed by one-way ANOVA, with multiple *post hoc* comparisons of Tukey's test for behavioral analysis or Bonferroni test for biochemical assays, considering a significance level of 95%. GraphPad Prism 7.0 software was employed to perform statistical analyzes and graphical building.

## 3. Results

### 3.1. AEGl Presents Low Toxicity

AEGl (1000 and 5000 mg/kg) administered acutely by oral route did not promote deaths during the acute oral toxicity assay period. However, such high doses resulted in sedative effects (Table 1), changes in oxidative balance (Table 2), and neutropenia (Table 3).

### 3.2. AEGl Recovers the Ability of Vertical Exploration of the Animals Previously Intoxicated by Ethanol in Pattern Binge in the of Test

**Figure 2:**
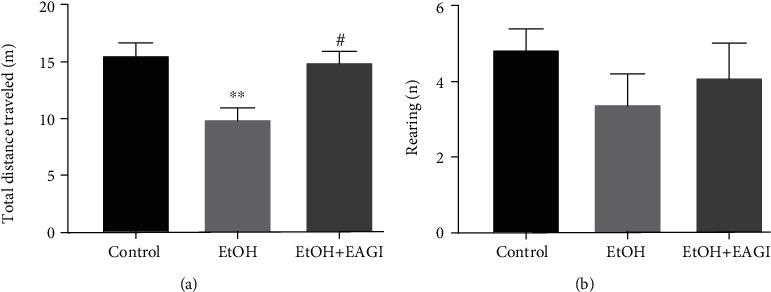
Effects of Aqueous Extract of *Ganoderma lucidum* (AEGl) on the spontaneous locomotion after the intoxication by ethanol in a binge pattern on adults' male rats. The analysis of the total distance traveled allowed to evaluate the horizontal exploitation of the animals (a), and the number of rearing as vertical exploitation (b), both related to motor activity of the animals. ^∗∗^*P* < 0.01 (compared to the control group); ^#^*P* < 0.05 (compared to EtOH group). Data were analyzed by One-way ANOVA (mean ± SEM; *n* = 7 − 10 animals per group).

To evaluate the activity of spontaneous locomotion in rodents, we can benefit from the natural tendency of animals of exploring new environments [33]. Motor injuries are a common impairment that occurs after ethanol intoxication in a binge pattern [9, 10]. For this evaluation, the OF test was performed. The AEGl (0.1 mL/100 g) recovered the reduction of total distance traveled displayed by binge drinking administration in rats (*P* = 0.0224, Figure 2(a)), in which the animals performed the horizontal ambulation similar to the control group. There was no significant difference between the groups in the evaluation of the rearing number (Figure 2(b)), which refers to vertical exploitation.

### 3.3. AEGl Improves Emotional Behaviors Resulted from the Consumption of Ethanol in a Binge Pattern

Ethanol is able of generating emotional disturbances [9, 10]. For the evaluation of emotional behaviors, i.e., anxiogenic- and depressive-type, the elevated plus maze (EPM) and forced swimming (FS) tests were performed, respectively. The former is based on the preference of the animals to dark, closed, and small places. Nevertheless, the animals still maintain the exploratory behavior [34]. The latter is known as a model of “behavioral desperation,” in which the depressive-like profile animals exhibit elevated immobility time during the task [27, 35].

**Figure 3:**
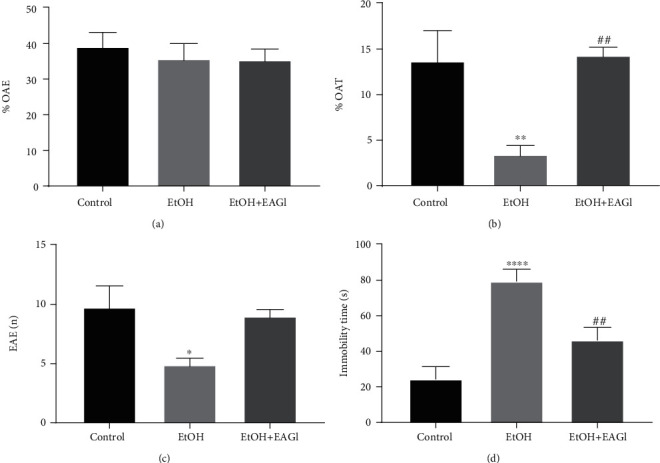
Effects of Aqueous Extract of *Ganoderma lucidum* on emotional alterations provoked by intoxication with ethanol (EtOH) in a binge pattern. The parameters percentage of entries in the open arms (a), percentage of time spent in the open arms (b), and the number of entries in the closed arms (c) allowed the assessment of the anxious behaviors and locomotor activity on the elevated plus-maze paradigm. Immobility time (d) parameters were admitted as an index of depressive-like behavior on forced swim test. ^∗^*P* < 0.05 (compared to control group); ^∗∗^*P* < 0.01 (compared to control group); ^∗∗∗∗^*P* < 0.0001 (compared to control group); ^##^*P* < 0.01 (compared to EtOH group). Data were analyzed by One-way ANOVA (mean ± SEM; *n* = 6 − 9 animals per group).

In the EPM test, animals that were submitted to the ethanol paradigm reduced the open arms time (%OAT; Figure 3(b)), which suggests the anxiogenic-like phenotype. Besides, the enclosed arms entries (EAE) parameter was reduced in the ethanol-exposed animals, which confirms the reduction on spontaneous motor performance (Figure 3(c)). Therefore, animals that received the *G. lucidum* extract increased the %OAT, reaching the levels related to the control group (*P* = 0.0067), as well improved motor ambulation that corroborates with the findings found in OF test.

In the FS test, the AEGl administration reduced the immobility time, displayed by successive binge drinking exposure in rats (Figure 3(d); *P* = 0.0110), which reflects antidepressant activity. After the mushroom extract intake, ethanol-treated animals reached immobility time values related to control animals.

### 3.4. AEGl Improves Short-Term Memory Damages Induced by Ethanol Binge Drinking Exposure

**Figure 4:**
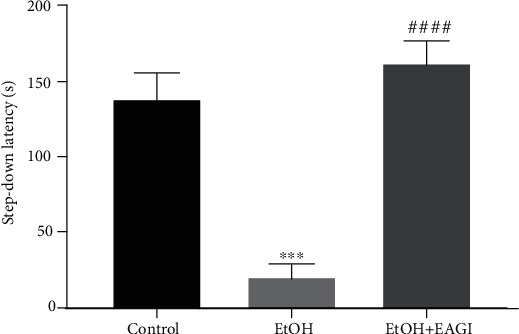
Effects of Aqueous Extract of *Ganoderma lucidum* (AEGl) on the short memory after the intoxication by ethanol (EtOH) in a binge pattern on adults' male rats. The step-down latency allowed to evaluate the short-term memory (1.5 h). ^∗∗∗^*P* < 0.001 (compared to control group); ^####^*P* < 0.0001 (compared to EtOH group). Data were analyzed by One-way ANOVA (mean ± SEM; *n* = 6 − 7 animals per group).

Studies have revealed that binge drinking leads to short-term memory impairment [9]. To evaluate the short-term memory, we applied the inhibitory avoidance test (IA) that consists of obtaining the behavioral response from an aversive stimulus (i.e., footshock) [36, 37]. One hour and a half after the training session that was applied to the aversive stimulus, animals were submitted to the short-term memory test. Animals that were exposed to the binge drinking protocol reduced the latency to descend from the secure platform. However, individuals that received AEGl recovered the ethanol-induced mnemonic damage, increasing the latency to step-down from the secure platform of the apparatus (*P* < 0.0001; Figure 4).

### 3.5. G. Lucidum Extract Promotes the Equilibrium of the REDOX Balance

REDOX balance is mediated by the equilibrium between the generation of reactive oxygen and/or nitrogen species and the amount of antioxidant defenses. The disturbance on such factors induces to severe damage in the organism [38]. In general, the AEGl treatment reestablished the REDOX balance, which promoted a counterbalance between free radicals and antioxidant defenses in ethanol-treated subjects.

**Figure 5:**
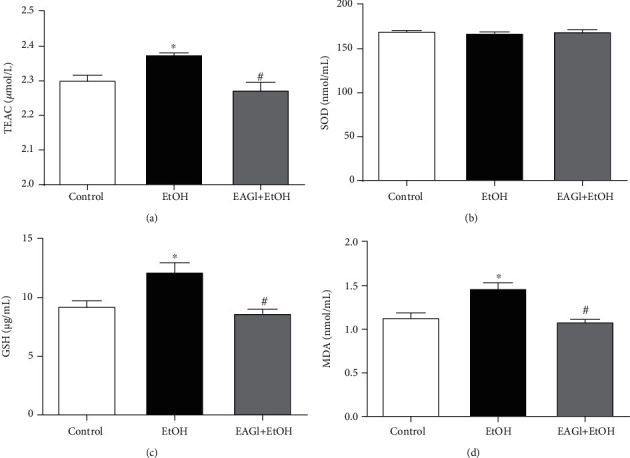
Effects Aqueous Extract of *Ganoderma lucidum* after intoxication by ethanol (EtOH) in a binge drinking pattern on the REDOX system. Total antioxidant capacity (TEAC; a); Superoxide dismutase (SOD) enzymatic activity (b); glutathione (c); and lipid peroxidation (MDA; d) were evaluated. ^∗^*P* < 0.05 (compared to control group); ^#^*P* < 0.05 (compared to EtOH group). Data were analyzed by One-way ANOVA (mean ± SEM; *n* = 5 animals per group).

Figures 5(a)–5(d) show that *G. lucidum* extract reduced lipid peroxidation, as well as induced the equilibrium of the antioxidant defenses, such as GSH and TEAC, that returned to the oxidative parameters related to the basal levels expressed by the control group. In recent years, our group has shown that exposure to ethanol leads to an overproduction of reactive species and levels of antioxidant factors, in an attempt to compensate for the oxidative stress that causes damage to macromolecules, such as increased lipid peroxidation in the body.

## 4. Discussion

This study provides the first evidence that AEGl, a functional food, ameliorates behavioral alteration on a model of psychiatric-like disorders induced by binge drinking paradigm. Our results demonstrated the recovery of the spontaneous horizontal exploration capacity of the animals in the OF test, as well as extinguished the anxiogenic- and depressive-profile induced by ethanol exposure. On the cognitive field, the administration of AEGl reversed the ethanol-induced short-term memory damage. The behavioral effects of the extract were associated to the reequilibrium of the animals' REDOX balance.

Firstly, we submitted animals to the toxicological test. OECD guideline 423 was applied to verify the acute oral toxicity of AEGl [39]. Our results revealed that the *Ganoderma* extract did not show any lethality in the doses of 2,000 and 5,000 mg/kg. According to OECD xenobiotics classification based on five levels of toxicity parameters scale [40], the AEGl toxicological findings establish the extract on the category 5, which reflects the low toxicity xenobiotic, since the median lethal dose (LD_50_) is over 5,000 mg/kg. The characterization of these safety standards endorsed their application in pharmacological tests and the security for consumption.

Studies on toxicity, ability to cause harmful effects on living organisms, and adverse effects of *G. lucidum* are uncommon [41]. Acute oral toxicity is the method used to assess the toxicity produced by a substance when administered within 24 hours, followed by a 14-day observation, used to check and classify the substances as to their lethality, establishing safety parameters for human health, evaluating in animals the neurobehavioral alterations [26, 42], as well as hematological [43] and biochemical parameters [44].

We adopted, for this evaluation, the fixed-dose procedure suggested by OECD 423 [39]. We highlight that excessive doses of the AEGl (over 2,000 mg/kg) may promote lethargy, oxidative unbalance, and hematological alterations, which emphasizes the care of the dose and time use of the extract.

After that, we employed our ethanol-induced neurobehavioral impairment protocol. Recently, we showed that ethanol reduced the spontaneous locomotion of the individuals submitted to the binge-drinking protocol during adolescence [9, 10]. Our results demonstrated that such an ethanol paradigm during the adult phase also induced the dulling of the exploitation. Regards emotionality, we also found that this same pattern display anxiogenic and depressant phenotype in adult rats. Our previous data on adolescent individuals have reported such psychiatric-like conditions [9, 10], however, this is the first time that we demonstrated that sequential binge drinking pattern (5 cycles of 3 on-4 off) during adolescence until early-adult life induces anxiogenic- and depressant-like behavior in rodents.

In addition to emotionality, ethanol intake also negatively affects the mnemonic process. In this sense, our data demonstrated the impairment of short-term memory through an aversive stimulus paradigm in adult animals that have been already proposed by our group during adolescence period [9].

Actually, acute exposure to ethanol modifies the neurotransmission patterns, which occurs the overstimulation of GABAergic pathways, due to high GABA_A_ receptor stimulation, as well as reduction of the glutamatergic pathway activity, related to the block of the NMDA glutamate receptors [45]. Besides, other neurotransmission pathways are also affected, i.e., serotonergic and dopaminergic, which result in alterations of behavioral responses [46, 47].

However, ethanol long-term exposure and withdrawal display compensatory physiological mechanisms to balance neurotransmissions, through negative regulation of GABA_A_ receptors, increased expression of NMDA receptors and loss of glutamate reuptake glial function, which results in overstimulation of CNS, known as excitotoxicity [46, 48–51].

Excitotoxicity leads to neuroglial activation and consequent neuronal death in the most varied CNS regions, culminating in the generation of reactive oxygen species (ROS), which in turn produces unbalance in the REDOX system and oxidative stress [52]. On the other hand, oxidative stress results in late inflammatory response due to oxidative damage, further increasing the production of ROS, which, in turn, accentuates the inflammatory process, that leads to chronic inflammatory and oxidative stress process [53, 54]. In fact, oxidative stress and inflammatory response are considered the main mechanisms involved in the damages caused by the ethanol consumption [1, 52, 54–56].

Ethanol exposure also leads to oxidative stress by other pathways, such as direct action on biological membranes of cells and organelles, i.e., mitochondria [52, 57]. Briefly, the lesion in the mitochondria membrane provokes dysfunction of the organelle, causing the overproduction of ROS and reduced production of ATP, which displays apoptosis [52, 57–59]. In addition, ethanol activates NADPH oxidase (NOX), which is highly expressed by glial cells, which provokes high concentrations of ROS in the CNS and neuronal cells death [1, 47, 52, 60]. Among the brain regions impaired, motor cortex, nuclei of the base, and cerebellum, that play a pivotal role on motor performance, can be highlighted, which reflects the poor motor performance by ethanol-intoxicated animals [4]. Besides the motor function, emotional behavior alterations are in accordance of damage on mesocorticolimbic system (i.e., ventral tegmental area, nucleus accumbens, amygdala, prefrontal cortex, and hippocampus), which leads to emotional disorders, i.e., depression and anxiety, as well as memory deficits [3, 54, 60–63]. In our results, animals treated with AEG1 displayed the behavioral profile related to control subjects on behavioral tasks, revealing that the mushroom has properties that counteract these damages generated by ethanol exposure.

In the findings on the REDOX balance in the ethanol-treated animals, we have evidenced an imbalance of this system, with an increase in ROS generation, culminating in oxidative stress due to lipid peroxidation, which could be observed in the MDA levels, a biomarker of oxidative stress that is indicative of damage to the plasma membrane [64, 65], that have been demonstrated by our group previously [9, 10]. On the other hand, animals treated with AEG1 obtained the normalization of the MDA levels, in which we hypothesize that the extract was able to reduce the damage on the biological membranes generated by the unbalance in the REDOX system after the consumption of ethanol. Other interesting data found in this study, that corroborate with the idea that AEG1 was able to reduce oxidative stress, are the results of GSH levels and TEAC. Such markers consist of antioxidant defenses.

The GSH, an important marker of cellular oxidative stress, has several antioxidant functions, serving as a cofactor of Glutathione Peroxidase (GPx) in the detoxification of hydrogen peroxide (H2O2), that in turn reduces reactive species [66, 67]. The TEAC, as already mentioned, measures total antioxidant capacity, which will include elements such as the Alpha-tocopherol, and that its main role is to inhibit lipoperoxidation reactions, being assisted by GSH [67]. We suppose that ethanol intoxication in the binge pattern elicits a significant elevation of GSH and TEAC levels due to an exacerbated physiological response in the attempt to compensate for the high production of reactive species and to reduce neuronal damage [54, 68]. Therefore, high production and consumption of these antioxidant markers can lead to failure of REDOX defense systems, culminating in severe oxidative stress [69]. In contrast, the AEGl was able to normalize these levels of antioxidants of the animals of the treated group, demonstrating that it elicits mechanisms to reduce oxidative damage, supporting that endogenous production of antioxidants defenses were controlled, remained at the same level of the control group.

In fact, some studies demonstrate that *G. lucidum* reduces oxidative damage and suppresses lipid peroxidation [56, 70]. Studies claim that this ability of *G. lucidum* is due to a large number of bioactive components present in the mushroom, such as ergosterol, flavonoids, unsaturated fatty acids, ganodermic acid (triterpene), and polysaccharides [70–74].

Polysaccharides are bioactive compounds that have been reported as an important neuroprotective molecule, acting as antioxidants [75]. The polysaccharides *β* (1,3) and (1,6)-D-glucans are the main components of these mushrooms [76]. In addition, a study demonstrated that animals treated with *G. lucidum* polysaccharide reduced apoptosis related to mitochondria in animal models of renal failure, demonstrating that this bioactive compound restores the mitochondrial function [58]. Besides, *G. lucidum* polysaccharide inhibited the translocation of p47phox, which consists of a neutrophil NADPH oxidase enzyme-forming subunit, responsible for the formation of the superoxide anion to the membrane, reducing the production of enzyme-dependent superoxide [57]. Finally, a study found that pretreatment with *G. lucidum* at a dose of 100 mg/kg promoted an improvement in energy metabolism and balance between inhibitory and excitatory neurotransmitters in the CNS of rats treated with ethanol [77], with reflects on behavioral profile.

Thus, we postulate that *G. lucidum* is able to reduce lipid peroxidation and consequent damages on the cells through oxidative stress mechanisms. In addition, we believe that occurs a reduction of the ROS generation by mitochondria after the treatment with the mushroom, due to its ability to improve the performance of mitochondrial activity, explaining the reduction of the requirement for compensatory exacerbated endogenous production of antioxidant defenses, returning to the levels of GSH and TEAC to baseline in the animals of the treated group.

In addition to the antioxidant activity, recent studies have shown that *G. lucidum* has immunomodulatory potential [78]. A study demonstrated that *G. lucidum* is able to inhibit TNF*α*, IL-1b, and IL-6 from cortical microglial cells, suppressing NF-*κ*B signaling [79]. It is also reported that *G. lucidum* polysaccharides can modulate the functions of many immune system components, such as antigen-presenting cells, T and B lymphocytes, NK cells, neutrophil granulocytes, dendritic cells, and cytokine production, demonstrating anti-inflammatory activity [72]. In this sense, we also believe that *G. lucidum* could also act by reducing neuroinflammatory responses.

Thus, we claim that *G. lucidum* promotes greater viability of the CNS cells, through reduced damage from oxidative stress and neuroinflammation, that consist of two important ethanol-induced deleterious effects mechanisms, which reflects in better performance of treated animals in all behavioral tests performed. In this way, we demonstrated that the mushroom has potential activities against the damages caused by alcohol consumption, characterizing the functional food as an important element that can be used as an alternative for the management of alcoholic disorders. Moreover, *G. lucidum* is a mushroom that has been used for years as a nutraceutical for the prevention or treatment of diseases of the most varied origin, which has the advantage of being used as a functional food. It is perceived here the timely viability of the use of this natural source as a food supplement to support on the management of alcohol-related disturbances as an adjuvant in therapeutics, since it presented low toxicity and interesting biological effects [74, 80, 81].

## Figures and Tables

**Table 1:** Behavioral screening after acute administration of Aqueous Extract of *Ganoderma lucidum* (AEG1) by gavage in adult female Wistar rats in the acute oral toxicity test. A = EAGl 2000 mg/kg; B = EAGl 5000 mg/kg; and C = control groups. (+) Presence of the behavior signals; (0) Absence of behavior signals.

Signal	Time after administration								
	30 min			1 h			2 h		
	A	B	C	A	B	C	A	B	C
Lethargy	+	+	0	+	+	0	+	+	0
Ptosis	+	+	0	+	+	0	+	+	0
Agitation	+	+	0	+	0	0	+	0	0
Tremors	0	0	0	0	0	0	+	0	0
Increase of grooming	0	+	0	0	0	0	0	0	0
Increase of rearing	0	+	0	0	0	0	0	0	0
Signal	Days after administration								
	Day 2			Day 4			Day 14		
	A	B	C	A	B	C	A	B	C
Lethargy	+	+	0	0	+	0	0	0	0
Mortality	0	0	0	0	0	0	0	0	0

**Table 2:** Analysis of the ratio between total antioxidant capacity (TEAC) and malondialdehyde (MDA) levels in animals treated with EAGl at doses of 2000 and 5000 mg/kg on days 1 and 14.

Group	Analyses period			
	Day 1		Day 14	
	TEAC/MDA	*P* value	TEAC/MDA	*P* value
AEGl (2000 mg/kg)	0.47 ± 0.08^∗^	<0.0001	0.41 ± 0.09^∗^	<0.0001
AEGl (5000 mg/kg)	0.44 ± 0.08^∗^	<0.0001	0.40 ± 0.04^∗^	<0.0001
Control	0.78 ± 0.08		0.71 ± 0.18	

**Table 3:** Analysis of hematological parameters in oral toxicity in the groups treated with EAGl at doses of 2000 and 5000 mg/kg and in the control group for 1 and 14 days. A = treated with EAGl 2000 mg/kg; B = treated with EAGl 5000 mg/kg; and C = control groups. Results expressed by mean ± SEM of 5-8 animals per group (one-way ANOVA, Tukey's test). ^∗^*P* < 0.0001 when compared to the control group.

Cells (%)	Analyses period					
	Day 1			Day 14		
	A	B	C	A	B	C
Lymphocytes	87.25 ± 7.63^∗^	79.75 ± 4.75^∗^	49.2 ± 3.44	84.7 ± 2.9^∗^	79 ± 8.7^∗^	52.1 ± 8.4
Neutrophils	11.25 ± 7.38^∗^	18 ± 4.5^∗^	44.6 ± 3.92	14.7 ± 3.1^∗^	15.7 ± 6.2^∗^	41.8 ± 7.9
Monocytes	1.5 ± 0.5	2.25 ± 0.88	3.2 ± 1.04	0.7 ± 0.4	3 ± 1.3	3.5 ± 0.7

## Data Availability

The behavioral and oxidative data used to support the findings of this study are available from the corresponding author upon request.
